# Sex-Specific Brain Deficits in Auditory Processing in an Animal Model of Cocaine-Related Schizophrenic Disorders

**DOI:** 10.3390/brainsci3020504

**Published:** 2013-04-10

**Authors:** Patricia A. Broderick, Taylor Rosenbaum

**Affiliations:** 1 Department of Physiology, Pharmacology & Neuroscience, The Sophie Davis School of Biomedical Education, The City College of New York, The City University of New York, New York, NY 10031, USA; 2 Doctoral Program, Department of Biology, The City University of New York Graduate Center, New York, NY 10016, USA; 3 Department of Neurology, NYU Langone Medical Center, NYU Comprehensive Epilepsy Center, New York, NY 10016, USA; 4 School of Arts and Sciences, Emory University, Atlanta, GA 30322, USA; E-Mail: tmrosen@emory.edu

**Keywords:** cocaine, schizophrenia, addiction, sexual dimorphism, mesocorticolimbic, nigrostriatal, neuronal circuits, pons, sensory-motor gating, psychostimulants, dorsocochlear nucleus, inferior, superior colliculi, acoustic nerve

## Abstract

Cocaine is a psychostimulant in the pharmacological class of drugs called Local Anesthetics. Interestingly, cocaine is the only drug in this class that has a chemical formula comprised of a tropane ring and is, moreover, addictive. The correlation between tropane and addiction is well-studied. Another well-studied correlation is that between psychosis induced by cocaine and that psychosis endogenously present in the schizophrenic patient. Indeed, both of these psychoses exhibit much the same behavioral as well as neurochemical properties across species. Therefore, in order to study the link between schizophrenia and cocaine addiction, we used a behavioral paradigm called *Acoustic Startle*. We used this acoustic startle paradigm in female *versus* male Sprague-Dawley animals to discriminate possible sex differences in responses to startle. The startle method operates through auditory pathways in brain *via* a network of sensorimotor gating processes within auditory cortex, cochlear nuclei, inferior and superior colliculi, pontine reticular nuclei, in addition to mesocorticolimbic brain reward and nigrostriatal motor circuitries. This paper is the first to report sex differences to acoustic stimuli in Sprague-Dawley animals (*Rattus norvegicus*) although such gender responses to acoustic startle have been reported in humans (Swerdlow *et al.* 1997 [1]). The startle method monitors pre-pulse inhibition (PPI) as a measure of the loss of sensorimotor gating in the brain's neuronal auditory network; auditory deficiencies can lead to sensory overload and subsequently cognitive dysfunction. Cocaine addicts and schizophrenic patients as well as cocaine treated animals are reported to exhibit symptoms of defective PPI (Geyer *et al.*, 2001 [2]). Key findings are: (a) Cocaine significantly reduced PPI in both sexes. (b) Females were significantly more sensitive than males; reduced PPI was greater in females than in males. (c) Physiological saline had no effect on startle in either sex. Thus, the data elucidate gender-specificity to the startle response in animals. Finally, preliminary studies show the effect of cocaine on acoustic startle in tandem with effects on estrous cycle. The data further suggest that hormones may play a role in these sex differences to acoustic startle reported herein.

## 1. Introduction

### 1.1. Cocaine

**Figure 1:**
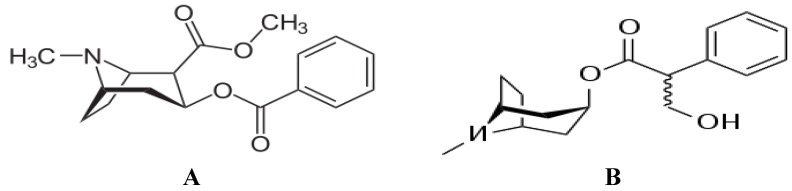
The figure depicts the cocaine molecule (**A**) and the atropine molecule (**B**) as seen in [3]. Note the tropane ring in bold. Atropine, although not a schedule II drug listed in the Drug Enforcement Agency Codes as is cocaine, is reported to be a “possibly addictive drug”. Atropine addiction occurs especially if it is used with another drug which has inherent addictive properties.

Cocaine is found in the leaves of the coca shrub; it is an ester of benzoic acid and methyl-ecgonine; ecgonine is an amino acid alcohol closely related to tropine, the amino alcohol of atropine. Atropine is a muscarinic anticholinergic agent used to block nerve impulses similarly to cocaine’s mechanism of action. In fact, atropine is sometimes called “possibly addictive”. However, to emphasize the role of cocaine as a local anesthetic, cocaine does have the same *fundamental* structure as the other classical synthetic local anesthetics given the caveat that its dramatic difference from the other local anesthetics lies in its “tropane” component (Figure 1). It is thought that tropane is the source of cocaine’s addictive properties. 

Cocaine blocks nerve impulses to produce local anesthetic properties. In this regard, cocaine possesses local vasoconstrictor properties secondary to inhibition of local norepinephrine reuptake; this enables cocaine to be the only local anesthetic that has its own vasoconstrictor properties. These unique properties make cocaine an effective local anesthetic. However, its cardiovascular effects and its proven potential for abuse have steadily decreased its clinical use [4].

Cocaine acts in the mesocorticolimbic neuronal pathway to produce euphoria, brain reward (*joie de vivre*)*.* Cocaine is a reinforcer and reinforcement is a cyclic phenomenon. Cocaine-induced euphoria is due at least in part to the inhibition of dopamine reuptake in mesocorticolimbic neuronal circuitry of brain and the dopamine transporter is believed to perform the mechanism by which dopamine remains in the synapse. Molecular pharmacology studies show that the tropane ring influences the reinforcing properties of cocaine [5,6]. 

Moreover, the reinforcing brain reward properties of cocaine are due, at least in part, to increased extracellular release of dopamine and serotonin in mesocorticolimbic circuitry, as detected by electron transfer with Neuromolecular Imaging (NMI). Also, with NMI, dopamine and serotonin antagonists e.g., atypical and typical antipsychotic agents were shown to block cocaine-induced release of monoamines while blocking psychostimulant behavior at the same time [7,8,9,10,11,12,13,14,15]. Similar effects of cocaine on extracellular levels of dopamine and serotonin using the method of microdialysis, are published [16,17,18,19].

### 1.2. Schizophrenia and Cocaine Psychoses

A study by Chapman *et al.* [20] brought about a view of schizophrenia which related schizophrenia to the process of cognition in the cerebral areas of attention and perception. Thus, the notion of schizophrenia as a “*breakdown of the ego*” [21] was revised. Schizophrenia is currently defined as a basic disorder of cognition [22,23,24], likely leading to sensory overload. To paraphrase what schizophrenic patients report as sensory overload, a schizophrenic patient may remark, “*too many things are coming into my mind*”; “*the thoughts feel bigger*”; “*the person I am talking to looks bigger*”. Therefore, the inability to gate sensory stimuli and channel the flow of sensory information to avoid sensory overload may well describe the schizophrenic/cocaine brain [25]. 

The hypothesis that correlates schizophrenic and cocaine psychoses is rooted in their neurochemical and behavioral similarities. Indeed, reports of cocaine-induced psychotic patients are happening at an alarming rate [26,27,28,29,30,31,32,33,34,35,36,37,38,39]. According to the mental health clinician’s handbook, *Diagnostic and Statistical Manual of* Mental Disorders, the broad category of cocaine-related disorders can be subdivided into cocaine use and cocaine disorders e.g. delusions (hanging on to beliefs that are clearly nonexistent) and hallucinations (hearing and seeing things) [40]. A clinical case of cocaine psychosis presenting with hallucinations and delusions, is reported [41]. This article reports a patient in the emergency department who had binged crack cocaine. Patient heard “voices in his head who were telling him to kill himself and the patient continued to believe there was a plot against him”. These auditory hallucinations have been given the definition, “strange perception of sounds” [42].

Thus, cocaine psychosis is a major psychopathology [43] and hyperfunction of dopaminergic systems is critical to drug-induced psychosis [44]. Too, what complicates the situation further, are data which show that about 50% of the patients who suffer from schizophrenia are also substance abusers at some time during their illness [45,46]. It is now known that substances with psychomimetic properties such as cocaine are used and abused worldwide and consumption of such substances can and will induce psychotic reactions including primary psychotic disease [47,48,49,50,51,52,53,54,55,56,57,58]. 

### 1.3. Responses to Auditory Stimuli, Startle and Pre-Pulse Inhibition (PPI)

Acoustic startle is said to, be protective [59,60], involve emotion [61], be aversive [62], and be used as a simple defensive reflex behavior [63]. This response or reflex is elicited by a sudden and intense acoustic stimulus. Facial and skeletal muscles are activated within a few milliseconds leading to a whole body flinch in rodents [59]. Although startle responses are reflective responses that can be reliably elicited, they are not stereotypic [60]. It is interesting to note that the startle response began with studies of the classical inhibitory tactile reflex and was further advanced by studying tactile reflexes in the sensory system of brain [64,65,66,67,68]. 

The term “pre-pulse inhibition” (PPI) was proposed by Ison [69]. Startle modification *via* auditory modulation of brain by startle became more interesting over ensuing decades due to its effects on sensorimotor gating [64]. Several psychiatric disorders, e.g., generalized anxiety disorder [70] and attention deficit hyperactivity disorder [71] are associated with deficits in sensorimotor gating and in particular, schizophrenic-related disorders are significantly affected by sensorimotor gating mechanism [72,73]. Normal PPI is a decrease in the startle amplitude in response to a strong sensory stimulus preceded 30–500 ms by a weak stimulus which is the “pre-pulse” [74]. It is important to note that it is a further reduction in PPI that leads to a brain deficit in the auditory system of the central nervous system. 

### 1.4. Auditory Stimuli-Startle Tests: Sex-Related Differences

Faraday and Grunberg performed animal studies on auditory stimuli wherein no drug was injected; instead the effects of proper acclimation times were studied and the use of proper acclimation times to reduce stress factors were emphasized [75]. The present paper is the first study of responses to auditory stimuli directly focused on sex differences and sex differences in the presence of cocaine in Sprague-Dawley animals. Thus, since cocaine and schizophrenia are known to be linked behaviorally and by neurochemical means, the purpose of this paper is to further study this link using a simple behavioral paradigm called *Acoustic Startle*. Moreover we explored the acoustic startle paradigm in male *versus* female Sprague-Dawley animals to discriminate possible sex differences in the auditory response to startle.

## 2. Experimental Section

### 2.1. Animals

Adult male and female Sprague-Dawley rats (2 months of age, weight matched for sex) were purchased from Charles River Laboratories, USA. Animals were housed in the Animal Care Facility under the auspices of the City College Institutional Animal Care and Use Committee (IACUC) in compliance with National Institute of Health (NIH) guidelines. Food (Purina Rat Chow) and water was available *ad libitum*.

Males were housed separately from females, three animals (male or female) per chamber. Studies were performed in groups of six, equal numbers of females and males during two seasons (Fall/Spring), with the exception of an additional group consisting only of six females, isolated from males during the experimental period. 

The auditory stimulus, acoustic startle response, termed pre-pulse inhibition (PPI), consisted of 4 trials, comprised of three tests, *Habituation*, *Injection* and *Post-injection*. The habituation period which was the first test was preceded by a 5 min acclimation period and each of the three tests was preceded by an acute injection of cocaine or physiological saline (animal weight in kilograms). The cocaine dosages administered were 10.0 mg/kg, 20.0 mg/kg and 40.0 mg/kg (dissolved in distilled water) and injected intraperitoneally (i.p.). 

The acoustic startle chamber is an SR-LAB™ model (San Diego Instruments, San Diego, CA, USA) automated to record the startle response of small animals. The chamber consisted of a cylindrical enclosure on a plexiglass base. Background noise, pre-pulse stimuli and startle stimuli were controlled by the SR-LAB™ software. Startle responses were transduced by a piezoelectric accelerometer mounted on a seismic sensor plate platform, located below the cylinder in which the animal is placed.

Startle response to auditory stimuli via muscle movements was studied by pre-pulse inhibition (PPI). Data were recorded as potential in voltage and were concatenated for compatibility with Excel software. %PPI was calculated using the following formula:

%PPI = 100 − [(mean PPI/mean startle only) × 100]

There were 25 sessions in each trial with each trial consisting of 3 tests; there was a 15 min interval between each trial. Each session consisted of 5 types which were randomly placed throughout the trial, but each trial used the same format, depicted below in tabular form (Table 1). 

**Table 1:** Protocol for Delivery of Auditory Stimuli in the Acoustic Startle Paradigm.

Type	No Stimulus	Prepulse	Pulse
1	75 decibels (broadband)	-	-
2	-	-	120 decibels
3	-	95 decibels	120 decibels
4	-	105 decibels	120 decibels
5	-	115 decibels	120 decibels

### 2.2. Data Analysis

Data were analyzed by Graph Pad Software Inc. and PRISM (La Jolla, CA, USA). Statistical significance between and within gender (sex) and dosage of cocaine was delineated via Two-Way Analysis of Variance (ANOVA). Data were considered significant at *p* < 0.05 and above. The expected baseline for acoustic startle response is defined by SR-LAB™ as 90th percentile.

## 3. Results

### 3.1. Sex Differences in Responses during the Acoustic Startle Paradigm after Cocaine

**Figure 2:**
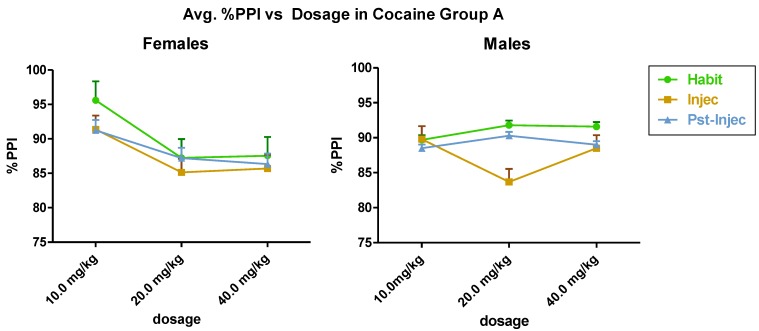
(Fall, Group A) (*n* = 6) shows the effect of cocaine on females (left) and males (right). Statistically significant differences occurred in the interaction between and within dosage.

**Figure 3:**
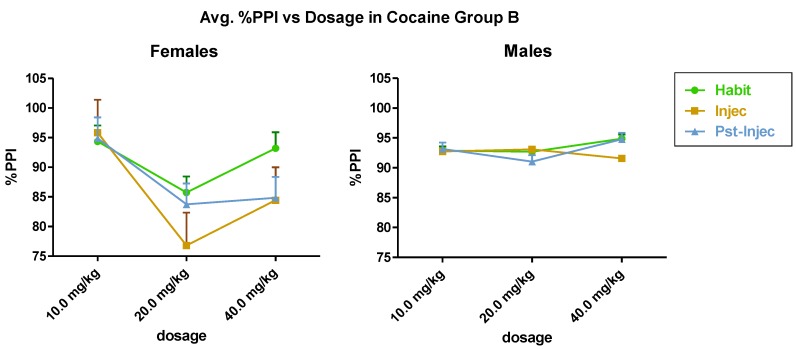
(Spring, Group B) (*n* = 6) shows the effect of cocaine on females (left) and males (right). Statistically significant differences occurred in the interaction between and within dosage and gender (sex).

Group A (Figure 2) showed statistically significant differences in the dose response interaction of cocaine in females and males (*F*(2,12) = 11.15, *p* < 0.002). In Group A, in females and males, the 20 mg/kg dose of cocaine showed a maximum effect in pre-pulse attenuation of acoustic startle. Group B (Figure 3) female *versus* male analysis showed dosage × gender interaction significance (*F*(2,12) = 6.81 and *p* < 0.01). Gender alone significance was analyzed, significance was found (*F*(1,12) = 11.90, *p* < 0.005). Cocaine dosage alone was significant as well (*F*(2,12) = 8.00, *p* < 0.006). In Group B, in females, the 20 mg/kg i.p. dose of cocaine resulted in a maximum attenuation of pre-pulse inhibition in the injection test. The within gender data gave the following results; Group A males *versus* Group B males showed between group significance, male × male (*F*(1,12) = 14.91, *p* < 0.002). Group A females with Group B females, females × females, showed cocaine dosage significance; (*F*(2,12) = 15.76, *p* < 0.0004).

**Figure 4:**
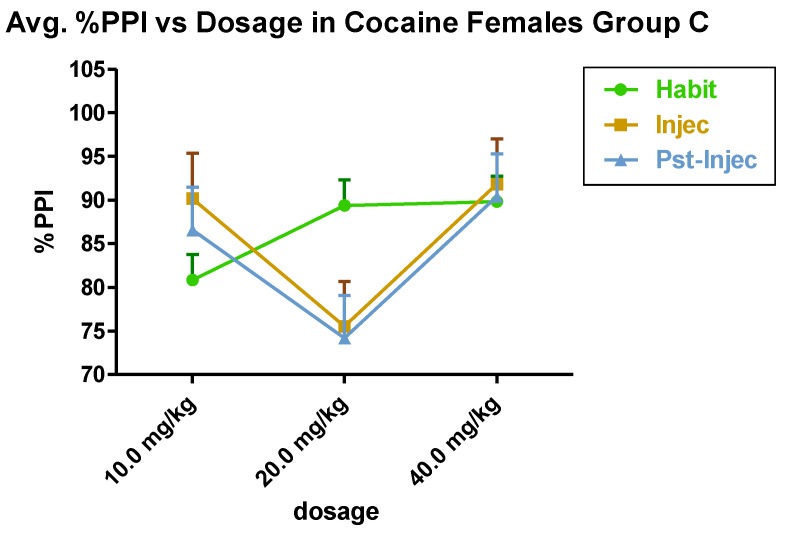
Group C (*n* = 6) shows the effect of cocaine in the acoustic startle paradigm in females. Females were isolated from males during the experimental period.

A significant dosage effect of cocaine in its attenuation of pre-pulse inhibition in Group C (isolated females) was seen at 20 mg/kg in the injection and post-injection tests in summation of 4 trials [95% Confidence Limits (p < 0.05)] (Figure 4). Given the caveat that different numbers of subjects comprised Group C and Group A females, still no female × female significance occurred. Nonetheless, Group C *versus* Group B, female × female comparison, exhibited a cocaine interaction significance in dosage (*F*(2,12) = 6.80, *p* < 0.01). 

### 3.2. Saline Control

**Figure 5:**
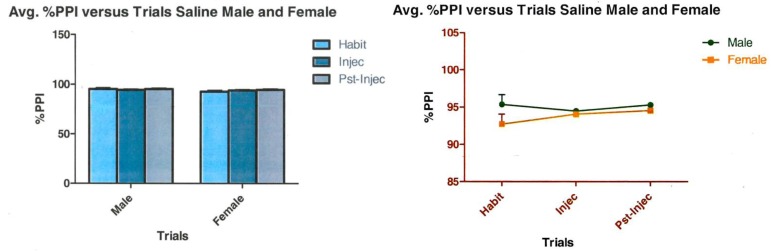
Shows the effect of saline administration to animals during acoustic startle.

The below line graph (Left)/bar graph (Right) is representative of an *n* value of 4 female and 4 male animals treated with saline (*n* = 8) (Figure 5). Analysis of the saline data shows an interaction between gender and trials that is not significant, (*F*(2,12) = 0.94, *p* < 0.42). This represents 10.63% of variance. Gender was not significant either, (*F*(1,12) = 3.42, *p* < 0.1). Gender was 19.27% of variance. Trials were not significant after administration of saline, (*F*(2,12) = 0.23, *p* < 0.80). Trials represent 2.55% of total variance. 

### 3.3. Summary of Groups A and B: Are There Seasonal Sex Differences in Acoustic Startle Responses?

Simply stated, the animals in the A group exhibited greater dosage interaction than sex interaction, whereas the animals in group B, exhibited significant dosage and sex differences. The studies were performed in different sets of animals seasonally. Nonetheless, whether or not the terminology, seasons, can be applied to the present studies, remains to be seen until further studies, using season as a variable, are explored.

## 4. Discussion

### 4.1. Sex Differences, Cocaine and the Estrous Cycle

Cocaine suppressed the pre-pulse inhibitory response to acoustic startle stimuli in females and males. The data confirm reports of cocaine’s ability to suppress pre-pulse inhibition in male animals ([76], see review [2]). This is the first report to show pre-pulse attenuation to acoustic startle by cocaine in female Sprague-Dawley animals. Females were more sensitive to startle than were males supporting results reported in female schizophrenic patients [77]. 

The data confirm previous studies that show sex differences to the effects of cocaine using a multiple of different methods [78,79,80,81,82]. Moreover, studies using the self-administration paradigm reported that *e*strogen plays a significant role in acquiring a desire for cocaine [83]. Ovarian hormones also play a significant role in the response to auditory stimuli via startle and stages of the estrous cycle stages have different responses to acoustic startle stimuli dependent upon the particular stage. In one study, pre-pulse inhibition was significantly reduced in the luteal as compared with the follicular phase of the menstrual cycle [84]. The suppression in the inhibitory response to auditory startle stimuli as measured by pre-pulse inhibition was most notable in the period corresponding to midluteal elevations in both estrogen and progesterone. A role for hormonal interaction during acoustic startle responses in females is suggested by preliminary studies in this laboratory. We performed tests of acoustic startle in concert with pre- and post-studies of estrous cycle stages, using vaginal smears. In these preliminary studies, administration of cocaine (20 mg/kg i.p.), changed the estrous cycle stage from metestrus to proestrus while pre-pulse inhibition in the same animal at the same time was dramatically suppressed. It is previously reported by others that estrous cycle stages are role players in cocaine addiction [85,86,87,88]. Strain differences are also critical to acoustic startle responses [85,86,87,88,89,90,91].

### 4.2. Pharmacological Manipulation of Startle

Dopamine, serotonin, somatostatin and neurotensin among others are involved in the startle response [92,93,94,95,96]. Importantly, depletions of neurotransmitters, receptor subtypes and genetic alterations of neurotransmitters provide excellent means to study acoustic startle mechanisms. Insofar as dopamine is concerned, D_1_R was found to be necessary for disrupting cocaine startle whereas D_2_R only partially contributes to the disruptive effects of cocaine-induced startle suppression [76]. Humby *et al.* [16] studied extracellular concentrations of dopamine in nucleus accumbens in male animals using the microdialysis method.

### 4.3. Brain Circuitry Mechanisms for the Auditory Startle Stimulus and Response

Rudomin in 2002 [97] reported that sensorimotor gating often occurs via presynaptic inhibition of the activated sensory neurons [97,98,99]. Neural circuits for acoustic startle to auditory stimuli have been postulated by Koch *et al*. [100,101,102,103,104], Fendt *et al.* [105] and Leumann *et al.* [106]. Neuronal links between ventral tegmental area and the causal pontine reticular nucleus, leading to a link between the mesocorticolimbic brain reward circuitry, and the pons was suggested by the Koch group. The Fendt group included the mesocorticolimbic and nigrostriatal pathway as participants in the acoustic startle circuitry. Leumann *et al.* proposed a major role for acetylcholine and glutamine for respectively inhibiting and exciting the acoustic startle response [106]. 

**Figure 6:**
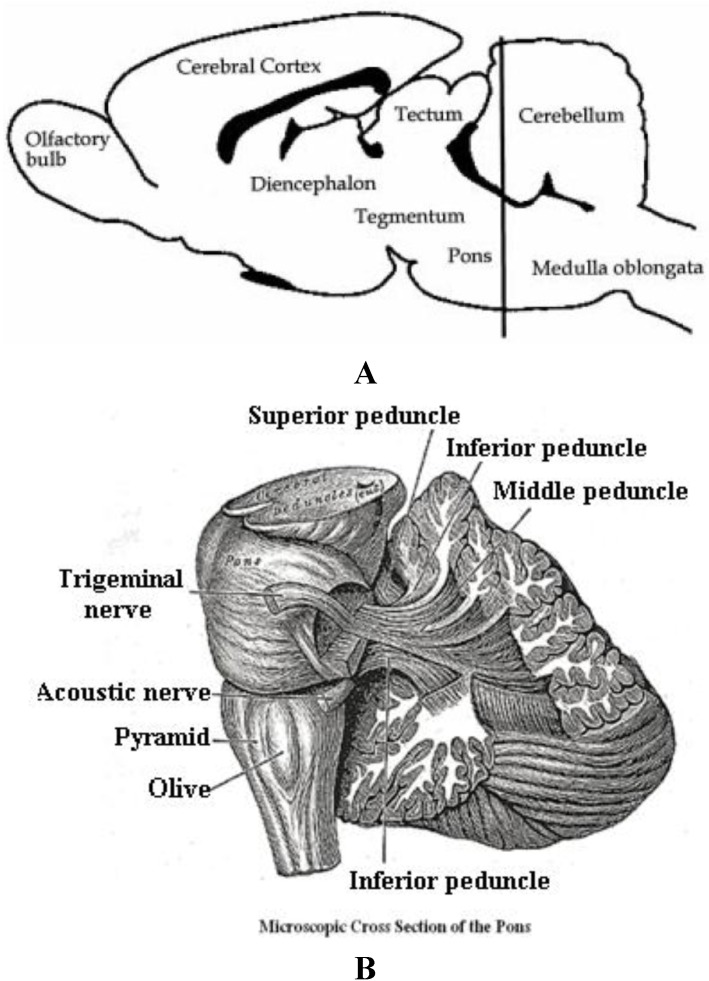
(**A**) Depicts a longitudinal section of murine and/or human brain that shows the position of the pons in relation to cerebellum, midbrain and forebrain structures. (**B**) Depicts a microscopic cross section of the pons in the human brain that shows the position of the acoustic nerve.

The brain stem is of the utmost importance since lesion studies show that the inferior and superior colliculi, the pons and the caudal pontine reticular nucleus are critically important in the execution of the startle response. Lentner and Cohen studied the role of the inferior colliculus in the startle reflex as early as the late nineteen eighties [107]. All these neuronal networks, whether studied empirically or postulated theoretically, underlie the mechanism of action for acoustic startle and all must rely on the anatomic brain structure called the pons, seen below in Figure 6A,B. 

Thus, the neural network underlying the mechanism of action of pre-pulse inhibition in the acoustic startle paradigm may include the nigrostriatal pathway, mesocorticolimbic circuitry linking the limbic cortex, striatum, pallidum and pontine reticular formation by monoamine, indoleamine, peptide and amino acid neurotransmitters. Of particular interest and germaine to this discussion is the close anatomic relationship between the ventrolateral tegmental nucleus and the pons. Such neuronal connections allow a rationale for associating emotions of reward and fear with the inhibitory and conversely, enhancing actions of pre-pulse inhibition during acoustic startle. Finally, further work is needed to delve into the neurochemical, hormonal and anatomical reasons for differences in the acoustic startle response in females and males. 

## 5. Conclusions

This is the first report of sex-specific differences in the acoustic startle paradigm in the presence of cocaine in Sprague-Dawley animals (*Rattus norvegicus*). The results show that cocaine attenuated pre-pulse inhibition in females and males; females exhibited greater attenuation in pre-pulse inhibition than did males. Statistically significant responses to acoustic startle were seen in females and males within and between sex (gender) and dosage regimens. A role for hormonal interaction during acoustic startle responses is suggested by preliminary tests of acoustic startle in concert with pre- and post-studies of estrous cycle stages. The data are important because new strategic inroads for sex-specific diagnosis and treatment of cocaine addiction and related schizophrenic diseases are clarified. 
